# Cys34-Cysteinylated Human Serum Albumin Is a Sensitive Plasma Marker in Oxidative Stress-Related Chronic Diseases

**DOI:** 10.1371/journal.pone.0085216

**Published:** 2014-01-08

**Authors:** Kohei Nagumo, Motohiko Tanaka, Victor Tuan Giam Chuang, Hiroko Setoyama, Hiroshi Watanabe, Naoyuki Yamada, Kazuyuki Kubota, Motoko Tanaka, Kazutaka Matsushita, Akira Yoshida, Hideaki Jinnouchi, Makoto Anraku, Daisuke Kadowaki, Yu Ishima, Yutaka Sasaki, Masaki Otagiri, Toru Maruyama

**Affiliations:** 1 Department of Biopharmaceutics, Graduate School of Pharmaceutical Sciences, Kumamoto University, Chuo-ku, Kumamoto, Japan; 2 Department of Gastroenterology and Hepatology, Graduate School of Medical Sciences, Kumamoto University, Chuo-ku, Kumamoto, Japan; 3 School of Pharmacy, Curtin Health Innovation Research Institute, Faculty of Health Sciences, Curtin University, Perth, Western Australia, Australia; 4 Center for Clinical Pharmaceutical Science, Kumamoto University, Chuo-ku, Kumamoto, Japan; 5 Institute of Innovation, Ajinomoto Co., Inc., Kawasaki-ku, Kawasaki, Japan; 6 Department of Nephrology, Akebono Clinic, Minami-Ku, Kumamoto, Japan; 7 Jinnouchi Clinic, Diabetes Care Center, Chuo-ku, Kumamoto, Japan; 8 Faculty of Pharmaceutical Sciences, Sojo University, Nishi-ku, Kumamoto, Japan; 9 DDS Research Institute, Sojo University, Nishi-ku, Kumamoto, Japan; Aligarh Muslim University, India

## Abstract

The degree of oxidized cysteine (Cys) 34 in human serum albumin (HSA), as determined by high performance liquid chromatography (HPLC), is correlated with oxidative stress related pathological conditions. In order to further characterize the oxidation of Cys34-HSA at the molecular level and to develop a suitable analytical method for a rapid and sensitive clinical laboratory analysis, the use of electrospray ionization time-of-flight mass spectrometer (ESI-TOFMS) was evaluated. A marked increase in the cysteinylation of Cys34 occurs in chronic liver and kidney diseases and diabetes mellitus. A significant positive correlation was observed between the Cys-Cys34-HSA fraction of plasma samples obtained from 229 patients, as determined by ESI-TOFMS, and the degree of oxidized Cys34-HSA determined by HPLC. The Cys-Cys34-HSA fraction was significantly increased with the progression of liver cirrhosis, and was reduced by branched chain amino acids (BCAA) treatment. The changes in the Cys-Cys34-HSA fraction were significantly correlated with the alternations of the plasma levels of advanced oxidized protein products, an oxidative stress marker for proteins. The binding ability of endogenous substances (bilirubin and tryptophan) and drugs (warfarin and diazepam) to HSA purified from chronic liver disease patients were significantly suppressed but significantly improved by BCAA supplementation. Interestingly, the changes in this physiological function of HSA in chronic liver disease were correlated with the Cys-Cys34-HSA fraction. In conclusion, ESI-TOFMS is a suitable high throughput method for the rapid and sensitive quantification of Cys-Cys34-HSA in a large number of samples for evaluating oxidative stress related chronic disease progression or in response to a treatment.

## Introduction

Post-translationally modified proteins can be used as biomarkers for the diagnosis of diseases or for the assessment of treatment responses. A prime example of this is the quantification of glycated hemoglobin and glycoalbumin for the diagnosis and treatment of diabetes mellitus [1], [2]. Recent studies have demonstrated an association between oxidative stress and the development of chronic diseases. There is growing interest in developing diagnostic tools to monitor the extent of oxidative damage in tissues or organs, and the use of novel anti-oxidants for the treatment or prevention of such oxidative stress-related diseases [3]–[5]. However, at present, a rapid and sensitive clinical laboratory testing method for the rapid assessment of oxidative stress in humans is lacking.

**Figure 1:**
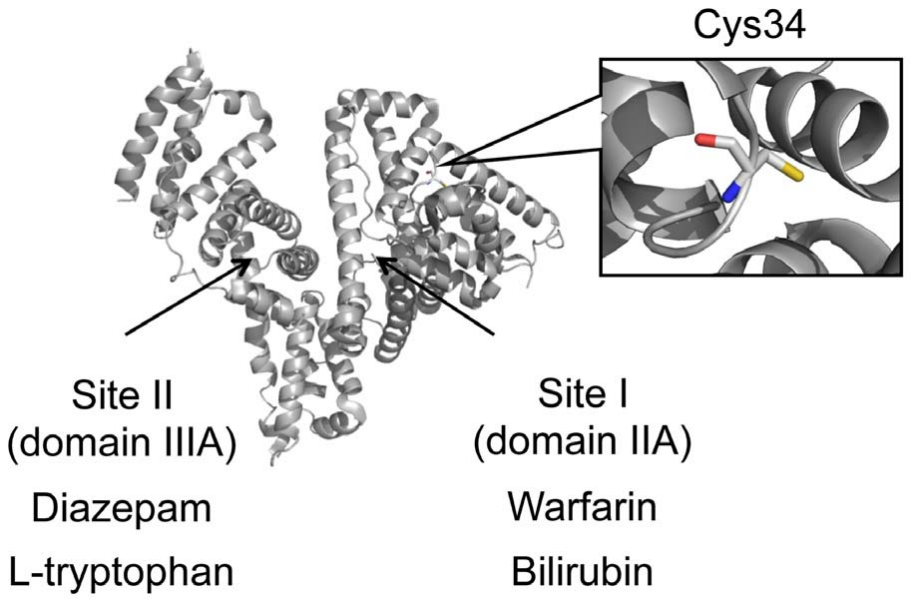
The structure of HSA. Superposition of the Cys34 and the location of two major ligand binding sites, Site I and II are shown.

Era's group and our laboratory have both developed a high performance liquid chromatography (HPLC) method to monitor the redox status of cysteine (Cys) 34 in human serum albumin (HSA) (Figure 1) [6], [7]. Since the redox status of thiol (SH) groups sensitively reflects the oxidation-reduction status of its surrounding environment, this HPLC analytical method has been shown to be useful for assessing the level of oxidative stress in diseased states and for evaluating the anti-oxidative activity of a therapeutic agent [8]–[11].

Previous studies of Cys34 have shown that Cys34 can be modified by treatment with cysteine, glutathione, homocysteine or drugs that contain a SH group. Cys34 can also be oxidized to a sulfonic acid and a sulfinic acid, but information on the degree of each modified form of Cys34 under diseased conditions is not available [12]–[16]. Unfortunately, the HPLC analytical method does not provide any molecular structural information regarding the oxidized form of Cys34. Therefore, we attempted to establish an analytical method that allows the quantitative and qualitative evaluation of the redox state of Cys34 with a high degree of sensitivity using electrospray ionization time-of-flight mass spectrometer (ESI-TOFMS) to analyze HSA that is cysteinylated at Cys34 (Cys-Cys34-HSA) in vitro using plasma samples obtained from healthy subjects [17], [18]. Moreover, recent analyses using ESI-TOFMS indicate that a significant amount of Cys-Cys34-HSA is present in the amniotic fluid of patients with gestational diabetes mellitus and in the plasma of dialysis patients [19], [20]. If Cys-Cys34-HSA accounts for the majority of the oxidized forms of Cys34, the fraction of Cys-Cys34-HSA, as estimated by ESI-TOFMS should correspond to the degree of oxidized Cys34-HSA determined by HPLC reported in previous studies, and this may permit the fraction of Cys-Cys34-HSA to be used as a novel marker for oxidative stress in the blood circulation.

Nevertheless, correlations between the fraction of Cys-Cys34-HSA determined by ESI-TOFMS and the degree of oxidized Cys34-HSA determined by HPLC have not been reported. Furthermore, the impact of the cysteinylation of Cys34 on HSA functions in a diseased state, and the progression of the disease or response to treatment remain unknown.

## Materials and Methods

### Chemicals

Blue Sepharose 6 Fast Flow was from GE Healthcare (Tokyo, Japan). Potassium warfarin (Eisai Co., Tokyo, Japan), L-tryptophan (Wako Pure Chemical Industries, Ltd., Osaka, Japan) and diazepam (Nippon Roche K.K., Tokyo, Japan) were obtained. All other chemicals were of the highest grade commercially available, and all solutions were prepared using deionized and distilled water.

### Ethics statement

The study was approved by the institutional review board of Kumamoto University, and each of the institutional review boards of the hospitals where samples were collected (“Ethics Committee of the Hospital Akebono Clinic, “Ethics Committee of the Hospital Jinnouchi Clinic”). All samples were obtained with written informed consent reviewed by the ethical board of the corresponding hospital.

### Clinical study of the correlation of Cys-Cys34-HSA and oxidized Cys34-HSA

9 mL blood samples were collected from individual study subjects and the samples were subjected to only one freeze thaw cycle, based on our previous work [18]. The collected blood was immediately diluted with 0.5 M sodium citrate buffer (pH 4.3) to stabilize for reduced albumin. Each plasma fraction was separated by centrifugation (4°C for 15 min, 2000 g). After collection, plasma samples were stored at −80°C until batch analysis.

#### Chronic liver disease

139 patients with liver cirrhosis who had been admitted to the Kumamoto University Medical School Hospital of Japan, between March 2011 and May 2013 were enrolled in this study. The stratified Child-Pugh class was used to estimate the severity of liver disease The liver cirrhosis patients were divided into 3 groups according to their Child-Pugh classification: Child-Pugh grade A (n = 79; 68.20±12.88 yrs), Child-Pugh grade B (n = 44; 62.71±11.93 yrs), and Child-Pugh grade C (n = 16; 54.73±7.27 yrs) (Table 1).

**Table 1:** General characteristics of the study participants.

	Healthy Subjects	Patients		
		Chronic Liver Disease	Chronic Kidney Disease (HD)	Diabetes Mellitus
**Category**	N = 9	N = 139	N = 38	N = 52
Gender (M/F)	6/3	60/79	20/18	21/31
Age (years)	61.4±4.4	57.4±15.0	58.6±16.1	59.0±16.1
Albumin (g/dl)	N/A	3.3±0.5	N/A	N/A
Bilirubin (mg/dL)	N/A	1.5±1.3	N/A	N/A
BUN (mg/dL)	N/A	N/A	63.6±11.8	N/A
SCr (mg/dL)	N/A	N/A	11.2±1.9	N/A
HbA1c (%)	N/A	N/A	N/A	7.9±1.6

Mean ± SD value are shown. BUN; blood urea nitrogen, SCr; serum creatinine, HD; hemodialysis.

#### Chronic kidney disease

38 patients with chronic renal failure who had been admitted to the Department of Nephrology of the Akebono Clinic of Japan, between March 2011 and May 2012 were enrolled in this study (58.55±16.06 yrs). End-stage renal failure in hemodialysis (HD) patients were caused by glomerulonephritis (n = 8), nephrosclerosis (n = 5) or diabetic nephropathy (n = 14) (Table 1). At the time of their enrollment, all of the HD patients were receiving regular bicarbonate HD therapy (4 to 5 hours per session, 3 times per week) using high-flux polysulfone hollow-fiber dialyzers.

#### Diabetes mellitus

52 patients with type 2 diabetes who had been admitted to the Jinnouchi Clinic, Diabetes Care Center of Japan, between March 2011 and May 2013 were enrolled in this study (58.98±16.06 yrs) (Table 1). Eligible patients were 21–86 years old, HbA1C>7% and suffering from type 2 diabetes for at least 1 year but no longer than 5 years.

#### Effect of branched-chain amino acid (BCAA) treatment on Cys-Cys34-HSA in chronic liver disease patients

60 patients with liver cirrhosis who had been admitted to the Kumamoto University Hospital of Japan, between March 2011 and May 2012 were enrolled in this study. The study population used for assessing the effect of BCAA treatment status is a sub-set of the patient population used for chronic liver diseases. These patients were given oral BCAA granules containing 952 mg of L-isoleucine, 1904 mg of L-leucine and 1144 mg of L-valine (LIVACT® Granules, AJINOMOTO PHARMACEUTICALS CO., LTD., Tokyo, Japan) at 4.15 g/sachet three times a day after meals. Plasma sample were collected at before, 3 and 5 months after treatment. The average albumin concentrations at before, 3 and 5 months after BCAA treatment were 2.85±0.48 g/dl, 3.21±0.43 g/dl and 3.36±0.40 g/dl respectively. Plasma samples from age and sex-matched nine healthy subjects as controls were obtained from Kumamoto University Hospital (Table 1).

### Determination of the degree of oxidized Cys34-HSA by HPLC

The SH redox status of HSA was determined with the HPLC system reported previously [8]. Briefly, 5 µL of aliquots of plasma were analyzed using a Shodex Asahipak ES-502N 7C column (Showa Denko, Tokyo, Japan). From the HPLC profile, the content of the oxidized form (HNA) and the un-oxidized form (HMA) of HSA was estimated as the area of each fraction divided by the total area of the plasma HSA peak. Using the levels of HNA and HMA, the degree of oxidized Cys34-HSA, (HNA/(HNA + HMA)) ×100, which is considered to be a reliable marker for the extent of systematic oxidative damage in uremic patients was estimated [10], [21].

### Solid phase extraction and ESI-TOFMS measurement of plasma samples

5 µL of plasma was added to 495 µL of 50 mM sodium phosphate buffer (pH 6.0). A solid phase extraction (SPE) column (Bond Elute-C18 EWP 200 mg/3cc, Varian, Inc., CA) was initialized with 10/90 water/acetonitrile containing 0.1% formic acid, then equilibration was performed with water (1 mL). The above-mentioned diluted plasma sample was applied to the equilibrated SPE column. The column was washed with 10% acetonitrile (2 mL) containing 0.1% formic acid, and albumin was eluted with 90% acetonitrile containing 0.1% formic acid.

2 µL of eluent was flow injected into the ESI-TOFMS (microTOF®; Bruker Daltonics Inc. USA) at a flow rate of 15 µL/min with 10/90 water/acetonitrile containing 0.1% formic acid using the auto sampler of Ultimate 3000 (Dionex, Idstein, Germany).

The data were acquired by the MicroTOF® software (Bruker-Daltonics) and processed for Maxent deconvolution using DataAnalysis® software (Bruker-Daltonics). The deconvolution mass range was set to be from 66000 to 68000 Da. And the mass peak of HSA and its modified molecules such as Cys-Cys34-HSA or glycated HSA, was automatically assigned and converted to an output text file using script with a resolving power of 10000 m/dm and absolute intensity threshold of 1000. The fraction of Cys-Cys34-HSA (%) was calculated by (Cys-Cys34-HSA/(Cys-Cys34-HSA + reduced HSA)) ×100.

### Purification of HSA from the plasma of chronic liver disease patients

HSA was purified from plasma samples using a previously reported method [21]. Briefly, HSA samples were isolated by Blue Sepharose 6 Fast Flow column (GE HealthcareTokyo, Japan). The samples were then dialyzed against deionized water for 48 h at 4°C, followed by lyophilization. The purity of the HSA samples was at least 95%, as evidenced by sodium dodecyl sulfate polyacrylamide gel electrophoresis (SDS-PAGE) and native-PAGE, respectively [21].

### Determination of the unbound fraction of L-tryptophan, warfarin or diazepam to purified HSA

The binding of L-tryptophan, warfarin or diazepam (12.5 µM) to HSA (25 µM) purified from the plasma of chronic liver disease patients and healthy subjects in 67 mM sodium phosphate buffer (pH 7.4) was examined by ultrafiltration at 37°C. The ultrafiltration was carried out using a YM-30 ultrafiltration device (Amicon/Millipore Inc., Bedford, MA, USA). HSA and the ligands were dissolved in 67 mM sodium phosphate buffer (pH 7.4). Samples of 250 µL were centrifuged at 10,000 *g* at 37°C for 3 min. Free (unbound) ligand concentrations in the filtrates were quantified by HPLC [22]–[24].

### Determination of the unbound fraction of bilirubin to purified HSA

The unbound bilirubin concentrations were determined using a modified horseradish peroxidase (HRP) assay [25]. 15 µM bilirubin was added to 30 µL HSA purified from the plasma of chronic liver disease patients and healthy subjects dissolved in 67 mM phosphate buffer (pH 7.4). 200 µL aliquots of the bilirubin/HSA complexes were placed in a 96 well-plate and the samples allowed to equilibrate for 20 min at 37°C. 10 µL of freshly diluted 1.75 mM hydrogen peroxide was then added to each well, followed by incubation for 3 min. The reaction was initiated by adding 10 µL of 1 ng/mL HRP. The rate of oxidative destruction of the unbound bilirubin was monitored by the reduction in the absorbance of the mixture at 450 nm for 10 min. The concentrations of unbound bilirubin were determined by extrapolating the rate of oxidation of bilirubin to a standard curve prepared by plotting the oxidation rate versus the concentration of bilirubin. It was assumed that only the unbound bilirubin was available as a substrate for HRP so that there was a linear relationship between the rate of destruction of bilirubin and the concentration of the unbound species.

### Determination of advanced oxidation protein products

Advanced oxidation protein products (AOPP) levels were determined spectrophotometrically by measuring the absorbance of the samples at 340 nm and expressed as chloramine-T equivalents after five-fold dilution of 200 µL of plasma with 20 mmol/L of pH 7.4 phosphate-buffered saline (PBS) and the subsequent addition of 80 µL of acetic acid, and reading against a blank PBS solution [26].

### Statistical analysis

All data were expressed as the mean ± SD. Differences between groups were examined for statistical significance using the Wilcoxon signed-rank test with Bonferroni correction, and the magnitude of each correlation were evaluated using Spearman rank-correlation coefficient. A P-value <0.05 denoted the presence of a statistically significant difference. The StatView 5.0 software was used for data analysis.

## Results

### Detection of Cys-Cys34-HSA in patients with chronic liver disease by ESI-TOFMS

**Figure 2:**
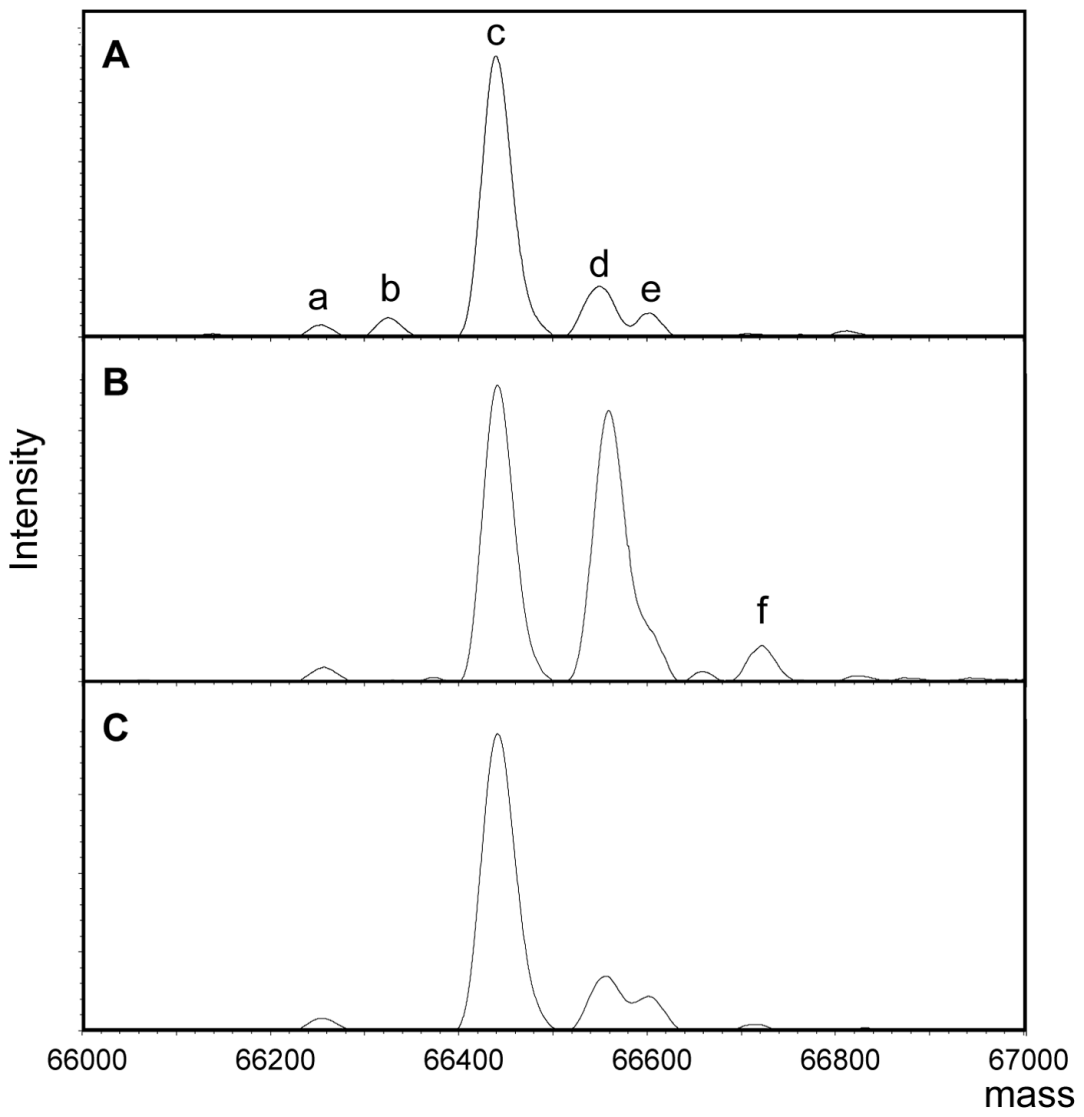
Deconvoluted ESI-TOFMS spectra of HSA. (A) Spectrum of HSA from healthy subject. (B) Spectrum of HSA from patient with chronic liver disease. (C) Spectrum of HSA from the same patient that (B) after DTT treatment. The peaks correspond to the following: (a) Asp-Ala truncation from N-terminal of HSA, (b) Leu truncation from C-terminal of HSA, (c) reduced HSA, (d) Cys-Cys34-HSA, (e) glycated HSA and (f) glycated Cys-Cys34-HSA.

Two major peaks, corresponding to Cys-Cys34-HSA and reduced Cys34-HSA, so-called HMA, were identified when plasma samples obtained from healthy subjects were analyzed by ESI-TOFMS (Figure 2A). We previously evaluated the reproducibility of the redox state of Cys34 measurement in human plasma. As a result, Cys-Cys34-HSA had high reproducibility values with a CV% of below 0.5% (intra-day, n = 4) and 1.55% (inter-day, n = 4) [18]. In agreement with previous findings, an increased Cys-Cys34-HSA peak accompanied by a decreased HMA peak was observed in patients with diabetes mellitus or chronic renal failure (data not shown) [19], [27]. Similar changes in the HSA mass spectra (MS) profile were also found for patients with chronic liver disease (Figure 2B). The HSA MS profile of chronic liver disease after dithiothreitol (DTT) treatment (Figure 2C) was similar to that for HSA profiles of healthy subjects (Figure 2A), indicating that the DTT treatment resulted in a reversal of the peaks for Cys-Cys34-HSA and HMA. These results confirm that the oxidative modification of Cys34 that occur in chronic liver disease patients are largely a reversible reaction, and that the most common oxidized form of Cys34 is cysteinylation.

### Correlation between Cys-Cys34-HSA and oxidized Cys34-HSA

**Figure 3:**
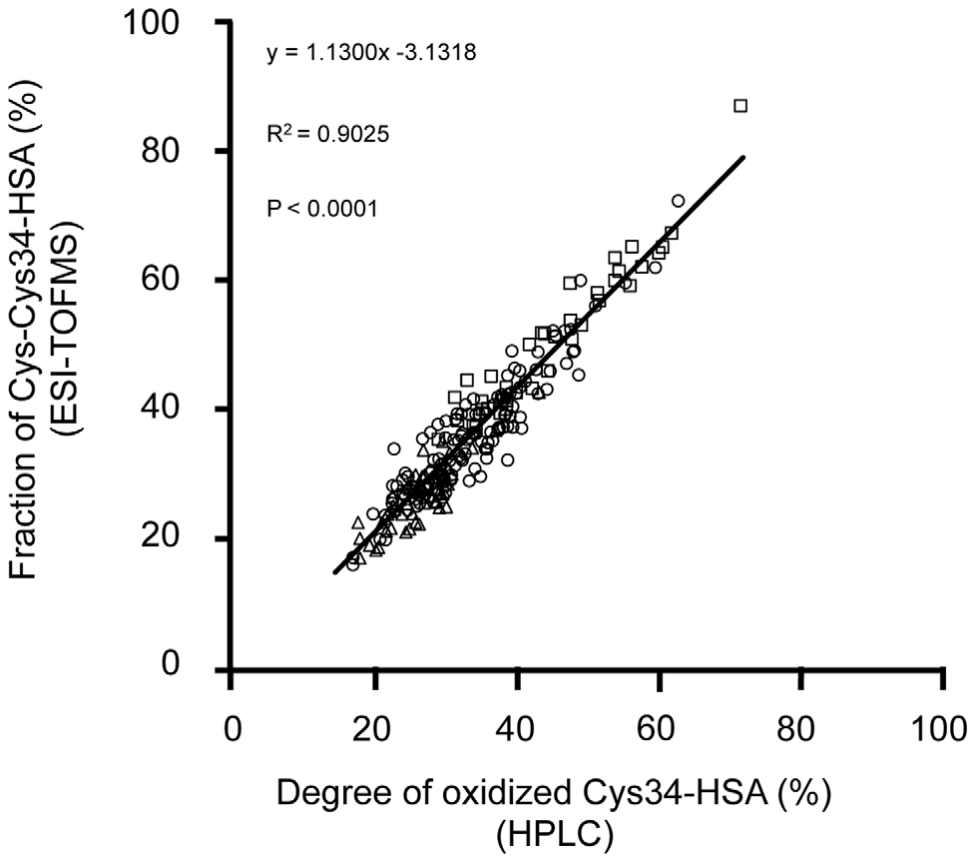
Relationship between the Cys-Cys34-HSA fraction (ESI-TOFMS) and the degree of oxidized Cys34-HSA (HPLC). The measured samples were 229 patients with chronic disease (including 139 patients with liver disease (circle), 38 patients with kidney disease (square) and 52 patients with diabetes mellitus (triangle)) (R^2^ = 0.9025, P<0.001).

The degree of oxidized Cys34-HSA and the Cys-Cys34-HSA fraction of plasma samples obtained from 229 patients with chronic disease (including 139 patients with liver disease, 38 patients with kidney disease and 52 patients with diabetes mellitus) was determined by HPLC and ESI-TOFMS respectively and the correlation between these two parameters was examined. As shown in Figure 3, a significant correlation was observed between the fraction of Cys-Cys34-HSA and the degree of oxidized Cys34-HSA. A significant correlation was also observed for each of the disease groups such as chronic liver disease, chronic kidney disease and diabetes mellitus (data not shown).

### Effect of disease severity or BCAA treatment on the Cys-Cys34-HSA fraction in patients with chronic liver disease

**Figure 4:**
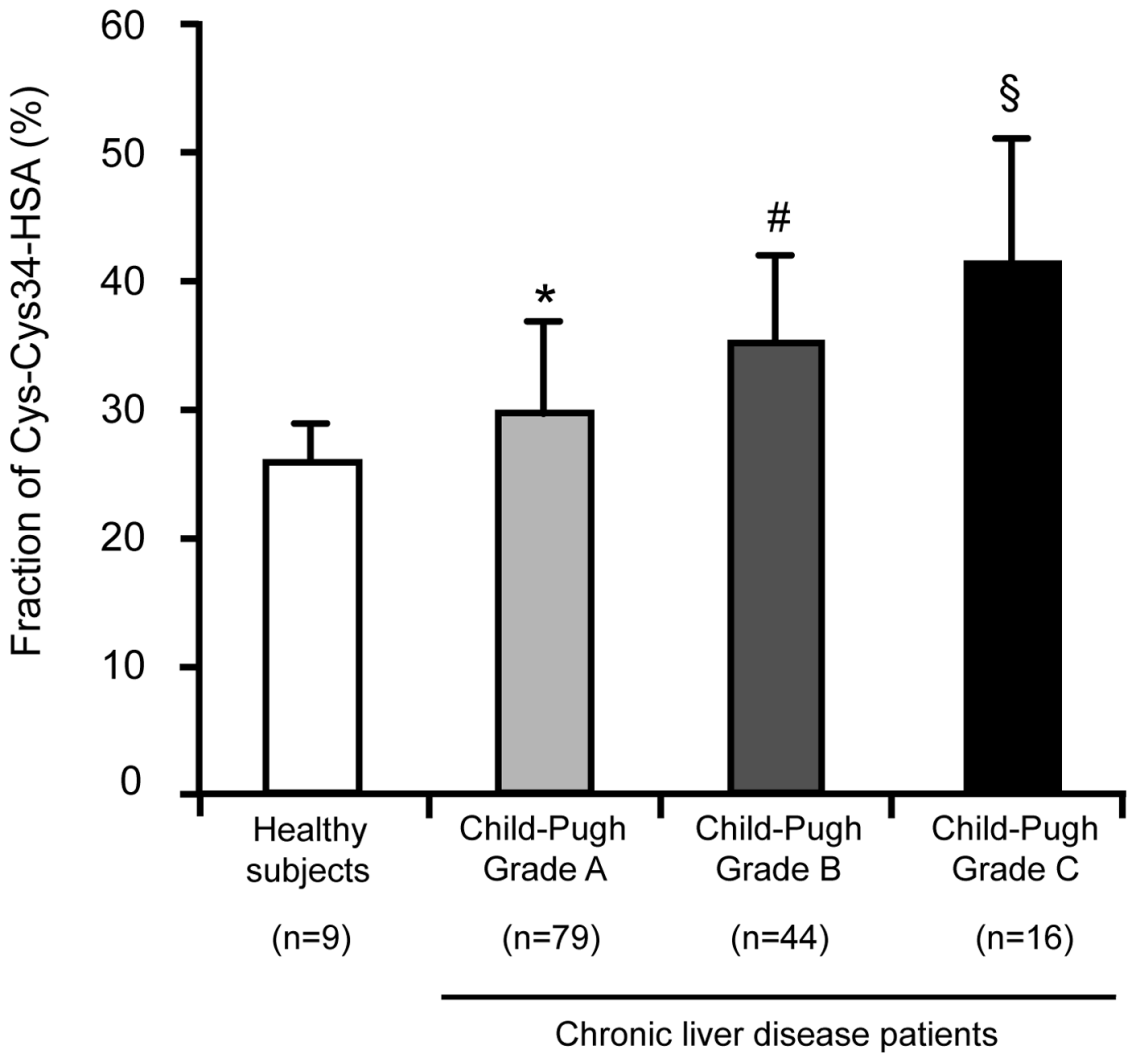
Effect of disease progression on the Cys-Cys34-HSA fraction in chronic liver disease patients. The Cys-Cys34-HSA fraction was measured by ESI-TOFMS. Values are expressed as mean ± SD (n = 9–79). *P<0.05 as compared with healthy subjects, ^#^P<0.05 as compared with Child-Pugh Grade A, and ^§^P<0.05 as compared with Child-Pugh Grade B.

The relationship between the Cys-Cys34-HSA fraction and Child-Pugh classification in patients with chronic liver disease was examined. As shown in Figure 4, an increase in the severity of the disease was associated with an increase in the fraction of Cys-Cys34-HSA, suggesting an association between the progression of chronic liver disease and oxidative stress.

**Figure 5:**
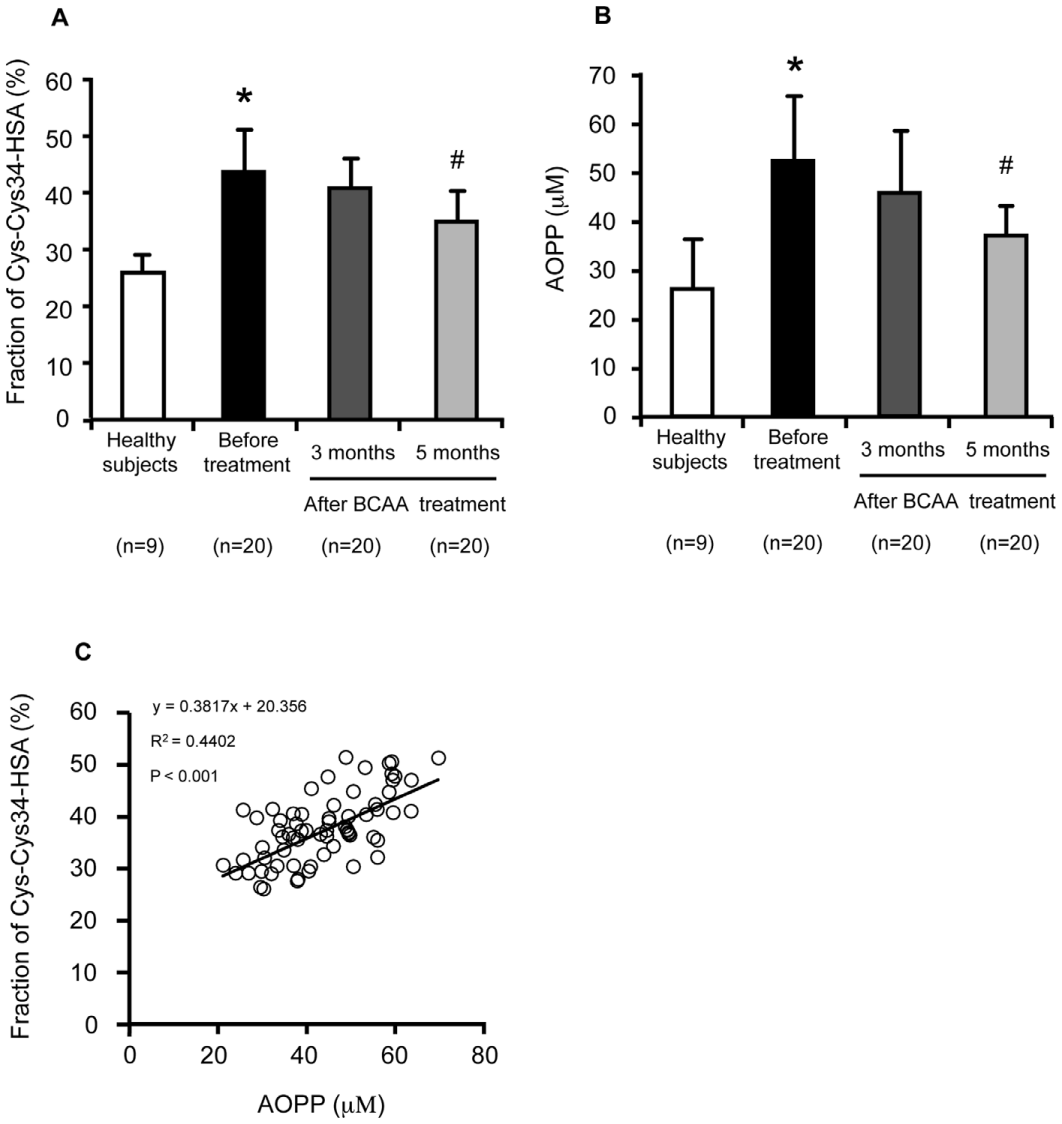
Change in the Cys-Cys34-HSA fraction and oxidative stress marker, AOPP, by BCAA treatment. (A) Change in the Cys-Cys34-HSA fraction. The Cys-Cys34-HSA fraction was measured by ESI-TOFMS. (B) Change in plasma AOPP level. (C) Correlation between the Cys-Cys34-HSA fraction and plasma AOPP level in chronic liver disease (n = 69). Values are expressed as mean ± SD (n = 20). *P<0.01 as compared with healthy subjects, ^#^P<0.05 as compared with before treatment.

The effect of BCAA treatment on the Cys-Cys34-HSA fraction in patients with chronic liver disease was examined. As shown in Figure 5A, the Cys-Cys34-HSA fraction was decreased as the result of the BCAA treatment, with a significant improvement compared to the pre-treatment level observed 5 months after the treatment. Figure 5B shows the effect of BCAA treatment on plasma AOPP levels, a marker of oxidative stress for proteins in patients with chronic liver disease. The plasma AOPP levels were also decreased as the result of the BCAA treatment at 5 months after the treatment (Figure 5B). The Cys-Cys34-HSA fraction was significantly correlated with plasma AOPP levels (Figure 5C), further supporting the conclusion that the Cys-Cys34-HSA fraction is, in fact, an appropriate marker for oxidative stress in the general circulation.

### Effect of the cysteinylation of Cys34 on ligand binding ability of HSA purified from the plasma of chronic liver disease patients

HSA has two major ligand bindings sites, so-called Site I and Site II where localized in subdomain IIA and IIIA on HSA, respectively (Figure 1). To investigate the effect of cysteinylation on ligand binding property, binding experiments by ultrafiltration technique were carried out using endogenous and exogenous substances. Bilirubin and L-tryptophan, which preferentially bind to Site I and Site II, respectively, were selected as models for endogenous ligands related to liver disease. On the other hand, warfarin and diazepam, representative drugs that bind to Site I and Site II respectively, were used as exogenous ligands.

**Figure 6:**
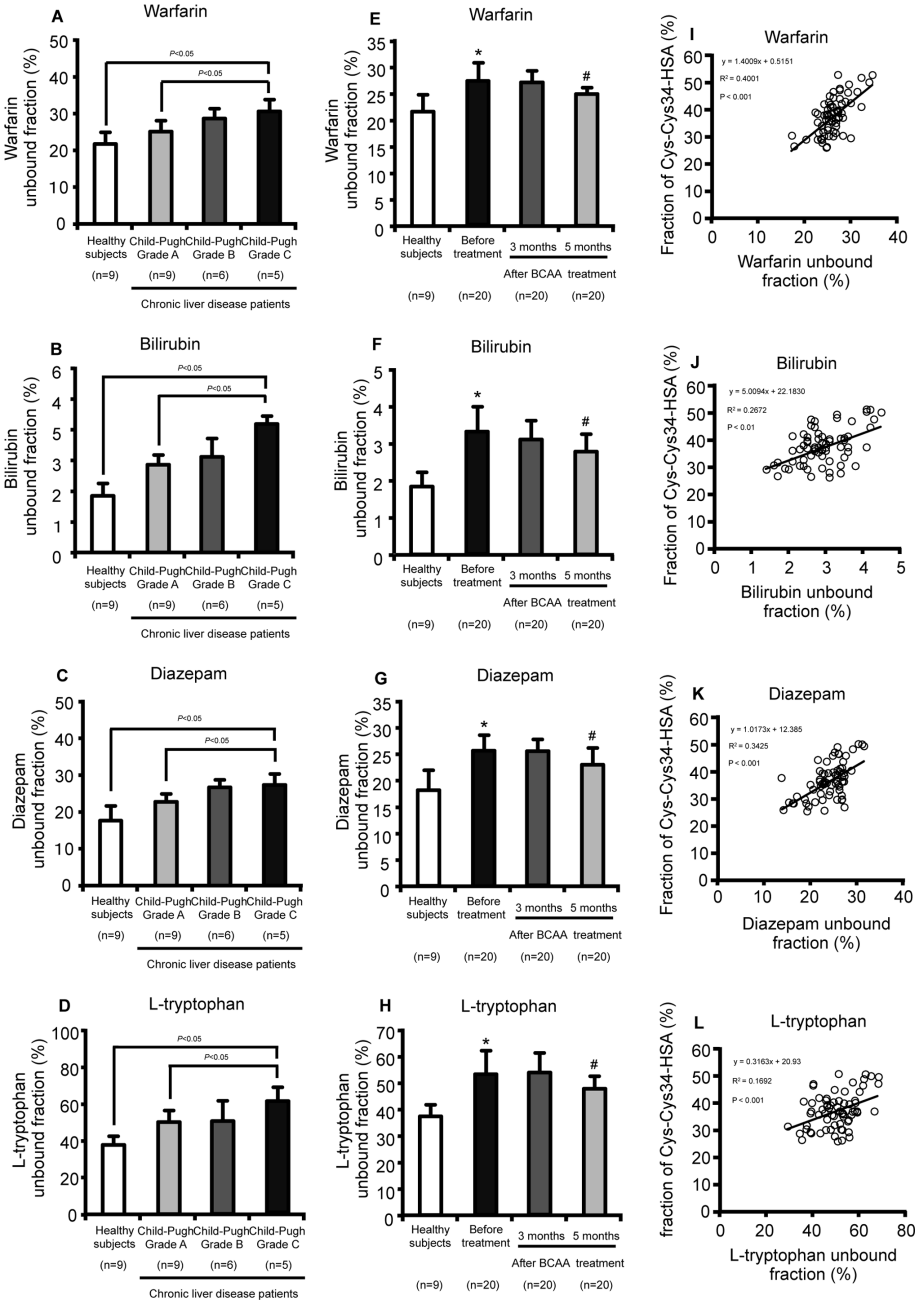
Relationship between the Cys-Cys34-HSA fraction and the ligand unbound fraction in chronic liver disease patients. Effect of disease progression on the unbound fraction of warfarin (A), bilirubin (B), diazepam (C) and L-tryptophan (D) to purified HSA. Effect of BCAA treatment on the unbound fraction of warfarin (E), bilirubin (F), diazepam (G) and L-tryptophan (H) to purified HSA. Correlation between the Cys-Cys34-HSA fraction and the unbound fraction of warfarin (I), bilirubin (J), diazepam (K) and L-tryptophan (L) to purified HSA in chronic liver disease. Values are expressed as mean ± SD. *P<0.01 as compared with healthy subjects, ^#^P<0.05 as compared with before treatment.

As reported previously, the HSA purified from the patients showed an increased unbound fraction for all four ligands compared to HSA purified from healthy subjects. As shown in Figure 6A–D, the increases in the unbound fraction of all of the four ligands were associated with the progression of liver disease. In contrast, as shown in Figure 6E–H, the decreases in the unbound fraction of all of the four ligands observed in patients with chronic liver disease were significantly improved by the BCAA treatment. Interestingly, a moderate correlation was observed for the unbound fraction of warfarin and diazepam and a causal relationship was shown in the case of the unbound fraction of bilirubin and L-tryptophan with the Cys-Cys34-HSA fraction (Figure 6I–L), suggesting that, the cysteinylation of Cys34 affected the microenvironment of the ligand binding sites.

## Discussion

Oxidative stress may cause the reversible and/or irreversible modification of sensitive proteins [17], [21], [28], [29]. Since the redox status of a free thiol group in proteins can be a very important indicator of oxidative stress, determining the redox status of Cys34 in HSA may allow the degree of organ damage by oxidative stress and the development of new anti oxidative therapeutic approaches. The utility of monitoring the redox status of Cys34 in HSA as a marker for oxidative stress in the circulation was demonstrated in a previous study by determining the degree of oxidized Cys34-HSA by HPLC [30].

However, in order to be able to examine alterations in the function of albumin by conventional procedures, the protein must be purified from at least several mL of plasma which may not be feasible in routine laboratory tests. Therefore, an analytical method that requires only a small amount of plasma under a clinical setting for the rapid measurement of the marker would be desirable. Compared to HPLC, ESI-TOFMS is a high throughput method that can be used to analyze large numbers of samples rapidly and sensitively, and thus may be suitable for use in clinical laboratory testing. Furthermore, the ESI-TOFMS method offers an additional advantage of having the capability of characterizing the different forms of Cys34 oxidized forms of Cys34 that are produced by oxidation by a variety of endogenous substances.

Using ESI-TOFMS, the present study provides evidence that the most common oxidized form is cysteinylated Cys34 (Figure 2B). As shown in Figure 3, a significant positive correlation with a slope of nearly 1 was observed between the degree of oxidized Cys34-HSA estimated by HPLC and the fraction of Cys-Cys34-HSA determined by ESI-TOFMS. These results suggest that the fraction of Cys-Cys34-HSA is a more sensitive marker for oxidative stress in plasma, than the degree of oxidized Cys34-HSA determined by HPLC. The present study is the first to show a marked oxidation, predominantly cysteinylation, of Cys34 in oxidative stress-related diseases such as chronic liver and kidney diseases and diabetes mellitus. The present study also found that the Cys-Cys34-HSA fraction increased in proportion to the Child-Pugh classification of cirrhotic patients. This is in agreement with the findings that oxidative stress plays an important role in the progression of chronic liver disease.

Recent studies have shown that the functional loss of HSA due to post-translational modification could influence homeostasis, which may contribute to the progression of chronic disease. Jalan et al. proposed that the loss of ligand binding capacity of albumin may be associated with an increased mortality of patients with decompensated cirrhosis [31]. Very recently, Oettl et al. reported that, in advanced liver disease, oxidative damage impairs the binding properties of HSA [32]. This reduced binding capacity of HSA is mainly related to impaired liver function. The decreased binding of toxic endogenous compounds to HSA may result in increased tissue distribution of these toxins, possibly enhancing the risk of tissue damage related to complications. In this study, a decreased binding affinity of bilirubin and tryptophan that might be related to liver disease was observed with increasing severity of liver disease. The present findings can be extrapolated to predict the binding of bile acids, another endogenous substance associated with disease progression, in chronic liver disease because bile acids bind Site I or Site II [33].

Interestingly, the changes in ligand binding were correlated with the alterations in the Cys-Cys34-HSA fraction. It is possible that the cysteinylation of Cys34 caused the changes in the microenvironment of the ligand binding sites, especially Site II, which could result in decreased ligand binding. On the other hand, the cysteinylation of Cys34 may further induce oxidative modifications to other amino acid residues that play an important role in ligand binding [17], [21]. Our previous in vitro examination showed that the cysteinylation of Cys34 could induce structural changes in the regions surrounding ligand binding sites [17]. Meanwhile, an increased cysteinylation of Cys34 was found to be accompanied by an increase in the carbonyl content of HSA [9], suggesting that other residues in addition to Cys34, especially arginine, lysine, histidine, tyrosine and tryptophan that significantly contribute to ligand binding to Site I and Site II are oxidized. In fact, we previously demonstrated that oxidative stress modified residues Lys195, Lys190, Arg218, Trp211 and Arg222 that are involved in the ligand binding to Site I, and Arg410 and Tyr411 residues, which play a crucial role in the binding of ligands to Site II [12]. Thus, both direct and indirect mechanisms appear to be responsible for the impaired ligand bindings accompanied with cysteinylation of Cys34.

BCAA treatment improves hypoalbuminemia in chronic liver disease patients by increasing the biosynthesis of HSA [34]. Recent studies have revealed that this effect is mediated by the activation of the mTOR pathway [35], [36]. The findings of this study indicate that the fraction of Cys-Cys34-HSA was significantly improved by BCAA treatment, most likely due to the increased biosynthesis of HMA. In addition to this indirect mechanism, recent studies have also demonstrated that BCAA per se has antioxidant activity [37]. It is thus possible that a reduction of oxidative stress caused by BCAA treatment led to a corresponding reduction in the cysteinylation of Cys34. On the other hand, the decreased binding affinity to bilirubin and tryptophan was recovered by a BCAA treatment, which is likely to be the result of an increased concentration of reduced Cys34 resulting from increased biosynthesis. Since the loss of ligand binding properties of HSA against these endogenous substances might contribute to an acceleration or inhibition of disease progression, the correlation between the Cys-Cys34-HSA fraction and the fraction of ligand binding to HSA suggests that the Cys-Cys34-HSA fraction might also serve as a marker for estimating changes in the physiological functions of albumin.

Recent studies revealed that albumin is an antioxidant in plasma and plays an important role in the homeostasis of the intravascular environment. Terawaki et al. reported that a low concentration of albumin with reduced Cys34 is associated with an increased incidence of severe cardiovascular disorders in dialysis patients due to a decreased antioxidative capacity [38]. This is because antioxidants are not as abundant in the plasma as they are in the cell, and albumin, which is abundant in plasma, serves as a dominant antioxidant in plasma. This is supported by the fact that about 80% of the total SH groups, a powerful ROS scavenger, present in plasma originate from Cys34 [15]. In a previous study, we reported that Cys34 accounted for approximately 40% of the total radical scavenging activity of HSA [39], [40]. Thus, the observed changes in the Cys-Cys34-HSA fraction with increasing severity of liver disease or BCAA treatment are likely to reflect an alternation in available SH groups resulting from the cyteinylation or de-cysteinylation of Cys34. Such changes in the cysteinylation of Cys34 could influence to the antioxidative capacity in general circulation, which may contribute to the progression of chronic disease.

### Conclusion

Cys-Cys34-HSA is a useful marker for estimating binding properties of HSA and monitoring oxidative stress in the plasma of chronic oxidative stress-related disease patients and ESI-TOFMS can be used in clinical laboratory testing.
